# The nod-like receptor, Nlrp12, plays an anti-inflammatory role in experimental autoimmune encephalomyelitis

**DOI:** 10.1186/s12974-015-0414-5

**Published:** 2015-10-31

**Authors:** Marjan Gharagozloo, Tara M. Mahvelati, Emilie Imbeault, Pavel Gris, Echarki Zerif, Diwakar Bobbala, Subburaj Ilangumaran, Abdelaziz Amrani, Denis Gris

**Affiliations:** Program of Immunology, Department of Pediatrics, CR-CHUS, Faculty of Medicine and Health Sciences, University of Sherbrooke, Sherbrooke, Quebec Canada; Montreal Neurological Institute, McGill University, Montreal, Quebec Canada

**Keywords:** *Nlrp12*, Experimental autoimmune encephalomyelitis, Microglia, Neuroinflammations

## Abstract

**Background:**

Multiple sclerosis (MS) is an organ-specific autoimmune disease resulting in demyelinating plaques throughout the central nervous system. In MS, the exact role of microglia remains unknown. On one hand, they can present antigens, skew T cell responses, and upregulate the expression of pro-inflammatory molecules. On the other hand, microglia may express anti-inflammatory molecules and inhibit inflammation. Microglia express a wide variety of immune receptors such as nod-like receptors (NLRs). NLRs are intracellular receptors capable of regulating both innate and adaptive immune responses. Among NLRs, Nlrp12 is largely expressed in cells of myeloid origins. It plays a role in immune inflammatory responses by negatively regulating the nuclear factor-kappa B (NF-κB) pathway. Thus, we hypothesize that Nlrp12 suppresses inflammation and ameliorates the course of MS.

**Methods:**

We used experimental autoimmune encephalomyelitis (EAE), a well-characterized mouse model of MS. EAE was induced in wild-type (WT) and *Nlrp12*^*−/−*^ mice with myelin oligodendrocyte glycoprotein (MOG):complete Freud’s adjuvant (CFA). The spinal cords of healthy and immunized mice were extracted for immunofluorescence and pro-inflammatory gene analysis. Primary murine cortical microglia cell cultures of WT and *Nlrp12*^*−/−*^ were prepared with cortices of 1-day-old pups. The cells were stimulated with lipopolysaccharide (LPS) and analyzed for the expression of pro-inflammatory genes as well as pro-inflammatory molecule secretions.

**Results:**

Over the course of 9 weeks, the *Nlrp12*^*−/−*^ mice demonstrated increased severity in the disease state, where they developed the disease earlier and reached significantly higher clinical scores compared to the WT mice. The spinal cords of immunized WT mice relative to healthy WT mice revealed a significant increase in *Nlrp12* messenger ribonucleic acid (mRNA) expression at 1, 3, and 5 weeks post injection. A significant increase in the expression of pro-inflammatory genes *Ccr5*, *Cox2*, and *IL-1β* was found in the spinal cords of the *Nlrp12*^*−/−*^ mice relative to the WT mice (*P* < 0.05). A significant increase in the level of gliosis was observed in the spinal cords of the *Nlrp12*^*−/−*^ mice compared to the WT mice after 9 weeks of disease *(P <* 0.05). Primary *Nlrp12*^*−/−*^ microglia cells demonstrated a significant increase in inducible nitric oxide synthase (iNOS) expression *(P <* 0.05) and secreted significantly *(P <* 0.05) more tumor necrosis factor alpha (TNFα), interleukin-6 (IL-6), and nitric oxide (NO).

**Conclusion:**

*Nlrp12* plays a protective role by suppressing inflammation during the development of EAE. The absence of *Nlrp12* results in an increased inflammatory response.

## Background

Multiple sclerosis (MS) is among one of the most common neurodegenerative diseases affecting an estimated 2.3 million individuals worldwide [1]. This organ-specific autoimmune disease is characterized by four different types of demyelinating plaques; types I and II which are T cell mediated or T cell and antibody-mediated, while types III and IV are mediated by oligodendrocyte death [2]. In all four cases, plaques are associated with activated macrophages, microglia, and astrocytes.

Regardless of the type of plaque formation, inflammation plays a central role in MS pathophysiology [1, 3].

Microglia, the resident immune cells of the central nervous system (CNS), play a major role in maintaining CNS homeostasis. They have been shown to be associated with developing plaques and are thought to contribute to the development of MS [2, 4], as well as other chronic inflammatory neurodegenerative diseases such as Alzheimer’s [5]. During MS, activated microglia can play the role of antigen-presenting cells (APCs) and, therefore, skew T cell responses towards a T helper cell 1 (Th1) pro-inflammatory phenotype [1, 2, 6]. In addition, once activated, microglia upregulate the expression of pro-inflammatory molecules including but not restricted to tumor necrosis factor alpha (TNFα), interleukin (IL)-1β, IL-6, macrophage inhibitory protein 1 alpha (MIP1α), and inducible nitric oxide synthase (iNOS), all of which have been shown to play a role in demyelination and neuronal damage [7].

There is a wide variety of immune receptors expressed by microglia that regulate its function. Pathogen-recognition receptors (PRRs) such as nod-like receptors (NLRs) are innate immune receptors and sensors of pathogen-associated molecular patterns (PAMPs) [8]. NLRs are a group of proteins that share a NACHT and leucine-rich repeat (LRR) domain but differ in their N-terminal effector domain. Upon recognition of their respective ligand, NLRs become activated and it result in the subsequent triggering of multiple pro-inflammatory molecular pathways, such as nuclear factor-kappa B (NF-κB). In addition, they are able to regulate both innate and adaptive immune responses and play a role in pathological processes [8]. Recently discovered *Nlrp12* is a pyrin-containing intracellular NLR protein. It is largely expressed in the cells of myeloid origin such as monocytes and dendritic cells (DCs). The expression of *Nlrp12* has been shown to play an important role in immune inflammatory responses by negatively regulating the NF-κB pathway and modulatory roles, such as dendritic cell migration [9, 10]. The NF-κB pathway is one of the major pathways involved in the inflammatory response. Typically, the activation of NF-κB following insults results in the transcription of pro-inflammatory cytokines such as TNFα, IL-1β, and IL-6; chemokines such as CCL5, CCL22, and MIP1α; and proteins, such as iNOS and cyclooxygenase 2 (COX2) [11, 12].

This study aims to investigate the role of NLRs in neuroinflammation, particularly to uncover the role of *Nlrp12* during experimental autoimmune encephalomyelitis (EAE) development. In our study, results show that Nlrp12 acts to downregulate inflammation during the development of EAE. This study may have significant implications in the development of potential novel therapies to treat MS and other neuro-inflammatory degenerative diseases.

## Materials and methods

### Mice

*Nlrp12* knock-out *(Nlrp12*^*−/−*^*)* mice were kindly provided by Dr. Jenny P. Y. Ting (Chapel Hill, NC). All of the protocols and procedures were approved by the University of Sherbrooke at the University of Sherbrooke Animal Facility and Use Committee.

### Experimental autoimmune encephalomyelitis

EAE was induced in 8–10-week-old C57BL/6 female mice using a previously established protocol by Miller et al. [13]*.* Briefly, a 1:1 emulsion mixture of myelin oligodendrocyte glycoprotein (MOG_35−55_) (Genemed Synthesis Inc., San Antonio, TX) and complete Freund’s Adjuvant (CFA) (Sigma-Aldrich, St. Louis, MO) supplemented with 100 μg *Mycobacterium tuberculosis* H37 RA (Difco Laboratories, Detroit, MI) was prepared using a glass tuberculin syringe. The MOG:CFA emulsion (100 μL) was injected subcutaneously on each side of the midline on the lower back of each mouse for a total of 200 μg MOG_35–55_ and 500 μg *Mycobacterium*. Pertussis toxin (200 ng) (List Biological Laboratories Inc., Campbell, CA) was injected intraperitoneally on the day of and 48 h following immunization. The mice were monitored every day for the development of disease. Clinical scores were given by two independent observers, using the following scale: 0, no sign of disease; 1, limp tail or weakness in limbs; 2, limp tail and weakness in limb; 3, partial limb paralysis; 4, complete limb paralysis.

### Histopathology

The immunized mice were anesthetized by intraperitoneal injection of Avertin® (2,2,2-tribromoethanol, approximately 240 mg/kg) (Sigma-Aldrich, St. Louis, MO) diluted in 0.9 % saline solution. The mice were then perfused with ice-cold phosphate-buffered saline (PBS) (Wisent, St. Bruno, QC), and the spinal cords were removed and stored at −80 °C immediately for RNA extraction (thoracic region) and placed in 4 % paraformaldehyde (Sigma-Aldrich, St. Louis, MO) for immunofluorescence analysis (lumbar region). The spinal cord tissues were embedded in paraffin and cut into 5-μm sections.

### T cell proliferation assay

T cell proliferation was performed using 3H-thymidine incorporation assay. A single cell suspension was prepared from draining the lymph nodes (more precisely, from the inguinal and axillary lymph nodes) and spleen. CD4^+^ T cells were then purified using EasySep Mouse CD4^+^ T Cell Isolation Kit (Stem cell, Vancouver, BC), seeded in a round-bottom 96-well culture plate (1 × 10^5^ cells/well) and activated with plate-bound anti-CD3 (1 μg/mL) and anti-CD28 (2 μg/mL) antibodies for 3 days. During the last 18 h of culture, 1 μCi of methyl-[3H]-thymidine (NEN Life Sciences, Boston, MA) was added per well. The cells were harvested onto glass fiber filter mats, and the incorporated radioactivity was measured using Top Count® microplate scintillation counter (PerkinElmer, Wellesley, MA).

### Intracellular IL-4 staining for flow cytometry

The purified CD4^+^ T cells from the wild-type (WT) and *Nlrp12*^−/−^ mice were activated by plate-bound anti-CD3 (1 μg/mL) and anti-CD28 (2 μg/mL) antibodies for 3 days. Then, the cells were stimulated with phorbol 12-myristate 13-acetate (PMA; 50 ng/mL, Sigma Chemical Co., St. Louis, MO) and ionomycin (1 μg/mL, Calbiochem Corp., La Jolla, CA) for 4 h at 37 °C and 5 % CO_2_ in the presence of Brefeldin A (1 μg/mL, eBioscience, San Diego, CA). After staining the cells with anti-CD4-FITC antibody (eBioscience), the cells were fixed and permeabilized using intracellular fixation and permeabilization buffer (eBioscience) and stained with anti-IL-4-PE antibody, as per the manufacturer’s instructions. Sample analysis was performed with FACSCalibur, and data analysis was done using FlowJo Software (FlowJo, LLC, Ashland, OR).

### RNA extraction, cDNA synthesis, reverse transcription and real-time quantitative PCR

RNA from the spinal cords and lymph nodes were extracted using TRIzol reagent (Life Technologies Inc., Burlington, ON). The tissues were homogenized with sterile beads (Qiagen, Limburg, Netherlands) at a speed of 20 Hz for 2 min. Chloroform (200 μL) (Fisher Scientific, Ottawa, ON) was added to each tube per 1 mL of TRIzol and incubated at room temperature for 15 min followed by centrifugation at 13,000 rpm for 15 min at 4 °C. Supernatants were collected in new tubes, and 500 μL isopropanol (Fisher Scientific, Ottawa, ON) was added to each tube and incubated for 10 min at −80 °C before spinning down at 13,000 rpm for 10 min at 4 °C. Pellets were washed with 75 % ethanol and re-suspended in 20 μL RNAse-free sterile water (Wisent, St-Bruno, QC). cDNA was synthesized using Oligo(dT) primer (IDT, Coralville, IA), PCR Nucleotide Mix (GE Healthcare, Baie d’Urfe, QC), M-MuLV Reverse Transcriptase, M-MuLV Reverse Transcriptase Buffer (New England BioLabs, Whitby, ON), and RNasin Ribonuclease Inhibitor (Promega, Madison, WI). Reverse transcription PCR (RT-PCR) and quantitative reverse transcription PCR (RT-qPCR) were used to verify the expression of *Nlrp12*, *Mip3α*, *Cox2*, *IL-1β*, and *Ccr5* using Brilliant III Ultra-Fast SYBR Green QPCR Master Mix (Agilent Technologies, Santa Clara, CA). Primers (IDT, Coralville, IA) sequences were as follows: *Nlrp12*F: 5′-CCT CTT TGA GCC AGA CGA AG-3′, *Nlrp12*R: 5′-GCC CAG TCC AAC ATC ACT TT-3′, *Mip3α*F: 5′-CTC AGC CTA AGA GTC AAG AAG ATG-3′, *Mip3α*R: 5′-AAG TCC ACT GGG ACA CAA ATC-3′, *Cox2*F: 5′**-**CCA GCA CTT CAC CCA TCA GTT-3′***,****Cox2*R: 5′-ACC CAG GTC CTC GCT TAT GA-3′**,***IL-1β*F: 5′-CAT CCA GCT TCA AAT CTC GCA G-3′, *IL-1β*R: 5′CAC ACA CCA GCA GGT TAT CAT C-3′, *Ccr5*F: 5′-CGA AAA CAC ATG GTC AAA CG-3′, *Ccr5*R: 5′-GTT CTC CTG TGG ATC GGG TA-3′, *18S*F: 5′-CGG CTA CCA CAT CCA AGG AA-3′, and *18S*R: 5′-GCT GGA ATT ACC GCG GCT-3′.

The samples were normalized to the internal control 18S rRNA, and relative expression was calculated using the ΔΔC_T_ method [14].

### Immunofluorescence

Slides were de-paraffinized in xylene (EMD Millipore, Etobicoke, ON) and hydrated in 100, 95, and 70 % ethanol gradient. Antigen unmasking was performed at sub-boiling temperature for 10 min in 10 mM sodium citrate buffer pH 6.0 (Sigma-Aldrich, St. Louis, MO). Immunofluorescence was performed in Sequenza Slide Rack and Coverplate System (Ted Pella, Inc., Redding, CA). The slides were washed with 0.1 % Triton X-100 in PBS solution, blocked in 5 % fetal bovine serum (FBS) plus 0.1 % Triton X-100 in PBS for 1 h and incubated with primary antibody (1:1000) overnight at 4 °C. Secondary antibody (1:2000) incubation was done at room temperature for 2 h. The slides were mounted with DAPI Fluoromount-G (SouthernBiotech, Birmingham, AL), and photomicrograph pictures were taken with Retiga SRV Mono Cooled numerical camera attached to Zeiss Axioskop 2 Microscope. The pictures were stitched with Adobe Photoshop CS6, and stain density was quantified with Image-Pro Plus 6.0 (Media Cybernetics, Inc., Rockville, MD).

### Antibodies

Rabbit anti-glial fibrillary acidic protein (GFAP) antibody was purchased from Cedarlane (Burlington, ON). Rabbit anti-ionized calcium-binding adaptor molecule 1 (Iba1) antibody was purchased from Wako (Osaka, Japan). Alexa Fluor 488 AfinniPure Goat Anti-Rabbit IgG (H + L) was purchased from Jackson ImmunoResearch Laboratories Inc. (West Grove, PA).

The percentage of microgliosis and astrogliosis in the spinal cord and gray matter were calculated as follows:

$$ \mathrm{Percentage}\ \mathrm{of}\ \mathrm{gliosis}\ \left(\%\right) = \frac{\mathrm{Density}\ \mathrm{stain}\ }{\mathrm{Total}\ \mathrm{area}} \times \mathsf{100} $$

The percentage of microgliosis and astrogliosis in the white matter were calculated as follows:

$$ \mathrm{Percentage}\ \mathrm{of}\ \mathrm{gliosis}\ \mathrm{in}\ \mathrm{the}\ \mathrm{white}\ \mathrm{matter}\ \left(\%\right) = \left(\frac{\mathrm{Density}\ \mathrm{stain}\ \mathrm{of}\ \mathrm{spinal}\ \mathrm{cord}-\mathrm{Density}\ \mathrm{stain}\ \mathrm{of}\ \mathrm{gray}\ \mathrm{matter}}{\mathrm{Total}\ \mathrm{area}\ \mathrm{of}\ \mathrm{spinal}\ \mathrm{cord}-\mathrm{total}\ \mathrm{area}\ \mathrm{of}\ \mathrm{gray}\ \mathrm{matter}}\right) \times \mathsf{100} $$

### Primary cell culture

Cortices from 1-day-old pups were extracted and placed onto a 100-mm petri dish using aseptic techniques. Cortices were sliced with a commercial razor blade, further broken up with a rigorous up-and-down motion in 10 mL of medium, and filtered with a 70-μm filter. The cells were then plated onto a 100-mm petri dish and put in an incubator of 37 °C with 5 % CO_2_. Cell culture medium DMEM/F12 (Wisent, St. Bruno, QC) was supplemented with 10 % FBS (Invitrogen, Burlington, ON), 1 % penicillin-streptomycin solution (Wisent, St-Bruno, QC), 1 % L-glutamine solution (Wisent, St. Bruno, QC), 0.9 % sodium pyruvate solution (Wisent, St. Bruno, QC), 0.9 % MEM amino acid solution (Wisent, St. Bruno, QC), and 0.9 % amphotericin B solution (Wisent, St. Bruno, QC). The medium of the mixed glial culture was changed every 2 to 3 days. After 3 weeks, primary microglia cells were separated from astrocytes using EasySep CD11b positive selection kit following the manufacturer’s instructions (Stem cell, Vancouver, BC).

### Immunoblotting

Proteins were separated in 10 % polyacrylamide gels and transferred onto PVDF (Millipore, Etobicoke, ON) membranes. The membranes were blocked with PBS containing 10 % nonfat milk and 0.05 % Tween-20 (Sigma-Aldrich, St. Louis, MO). The membranes were washed in 1× Tris-buffered saline (TBS) plus 1 % Tween-20 for 15 min and incubated with primary antibody (1:1000) overnight at 4 °C and with secondary antibody (1:2000) for 2 h at room temperature. The membranes were revealed with GE HealthCare Life Sciences Amersham ECL Plus (Baie d’Urfe, QC) and viewed with Molecular Imager VersaDoc from BioRad, and protein bands were quantified using NIH ImageJ software. The antibodies used were as follows: β-actin (rabbit), iNOS (rabbit), and anti-rabbit IgG HRP-linked antibodies were purchased from Cell Signaling Technology (Beverly, MA).

### Cytokine measurement

TNFα and IL-6 cytokines in the supernatant of microglia culture were measured using ELISA kits purchased from BioLegend (San Diego, CA). Cerebellum and lymph node samples were homogenized in 0.5 mL of ice-cold lysis buffer (Cell Signaling Technology, Beverly, MA) supplemented with protease inhibitors (Roche Diagnosis, Mannheim, Germany) by rapid agitation for 2 min in the presence of 3-mm stainless beads. The tissue lysate was centrifuged for 10 min at 13,000×g in a cold microfuge, and the supernatant was transferred to a new tube. The concentration of proteins in the lysate was determined by Bradford protein assay. The tissue levels of IL-4 were determined using a high sensitivity IL-4 ELISA Kit (eBioscience, San Diego, CA), and the concentration of IL-4 in serum samples was quantified using Mouse IL-4 DuoSet (R&D Systems), according to the manufacturer’s instruction.

### Statistical analysis

All statistical analyses were conducted using GraphPad Prism 6 software. The results were expressed as mean ± SD. Statistical significance was determined using one-way ANOVA Kruskal-Wallis followed by Bonferroni (*EAE clinical score)*, one-way ANOVA followed by Tukey-Kramer (*Nlrp12 mRNA expression, iNOS expression in primary microglia, concentration of pro-inflammatory cytokines*), two-way ANOVA followed by Tukey’s (*percentage of gliosis*), or one-way ANOVA followed by Dunet (*pro-inflammatory mRNA expression*) multiple comparison test. IL-4 results were compared between WT and *Nlrp12*^−/−^ mice using Mann-Whitney *U* test. Statistical significance was accepted at *P* < 0.05.

## Results

### *Nlrp12* mRNA expression reaches a peak at the third week post injection

**Fig. 1:**
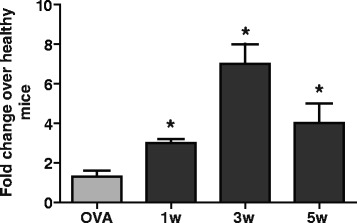
*Nlrp12* mRNA expression reaches a peak at third week post injection. Results indicate fold change in *Nlrp12* mRNA expression of the diseased mice over the healthy mice. The mice injected with ovalbumin as control were sacrificed 3 weeks post injection. Results are expressed as mean ± SD. Statistical significance was accepted at *P < 0.05. Statistical analysis was done using one-way ANOVA followed by Tukey-Kramer multiple comparison test. *n* = 5

Following immunization with ovalbumin and MOG_35–55_ in CFA, the spinal cords were dissected from healthy and EAE mice and analyzed for the expression of *Nlrp12* messenger ribonucleic acid (mRNA) (Fig. 1). *Nlrp12* mRNA expression in the immunized mice was shown to be significantly increased relative to the healthy wild-type (WT) mice at week 1 (threefold increase), week 3 (sevenfold increase), and week 5 (fourfold increase). Additionally, the level of *Nlrp12* mRNA expression was increased as of the first week of EAE and reached its highest level at the third week. At 5 weeks post injection, although the expression of *Nlrp12* was significantly higher in the diseased mice compared to the healthy mice, it was considerably lower than the third week and resembled much more the disease state of the first week. As a control, ovalbumin was injected, and the spinal cords of the mice treated with ovalbumin were removed after the third week in order to keep consistency with MOG-injected mice.

### *Nlrp12*^*−/−*^ mice exhibit exacerbated form of the disease compared to WT mice

**Fig. 2:**
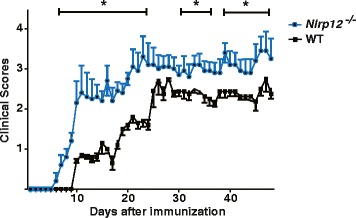
*Nlrp12* ^*−/−*^ mice exhibit an exacerbated form of disease compared to WT mice. Animals were scored daily by two independent observers and scored based on the following scale: 0, no sign of disease; 1, limp tail or weakness in limbs; 2, limp tail and weakness in limbs; 3, partial limb paralysis; and 4, complete limb paralysis. Statistical significance was accepted at *P < 0.05. Statistical analysis was done by Kruskal-Wallis one-way ANOVA test followed by Bonferroni multiple comparison test. *n* = 7

In order to investigate the role of *Nlrp12* in MS, EAE was induced in 8–10-week-old C57BL/6 female mice. An emulsified mixture of MOG_35–55_ in CFA was subcutaneously injected in mice. The *Nlrp12*^*−/−*^ mice demonstrated clinical symptoms after approximately 5 days post injection whereas the WT mice developed the disease roughly after 9 days. In addition, while the WT mice were showing the first signs of disease, the *Nlrp12*^*−/−*^ mice already demonstrated indications of severe disease, reaching scores of 2, indicative of tail and back limb weaknesses (Fig. 2). Indeed, the *Nlrp12*^*−/−*^ mice were observed to reach higher clinical scores throughout the 9-week period. More precisely, they reached scores of 3–3.5, which indicates weakness in the tail, back, and front limbs compared to the WT mice that reached scores of 2–2.5. In both genotypes, the severity of the disease outcome was observed to peak around the third week post injection and remained relatively constant throughout the 9-week period.

### *Nlrp12*^*−/−*^ mice demonstrate higher percentage of reactive gliosis after EAE

**Fig. 3:**
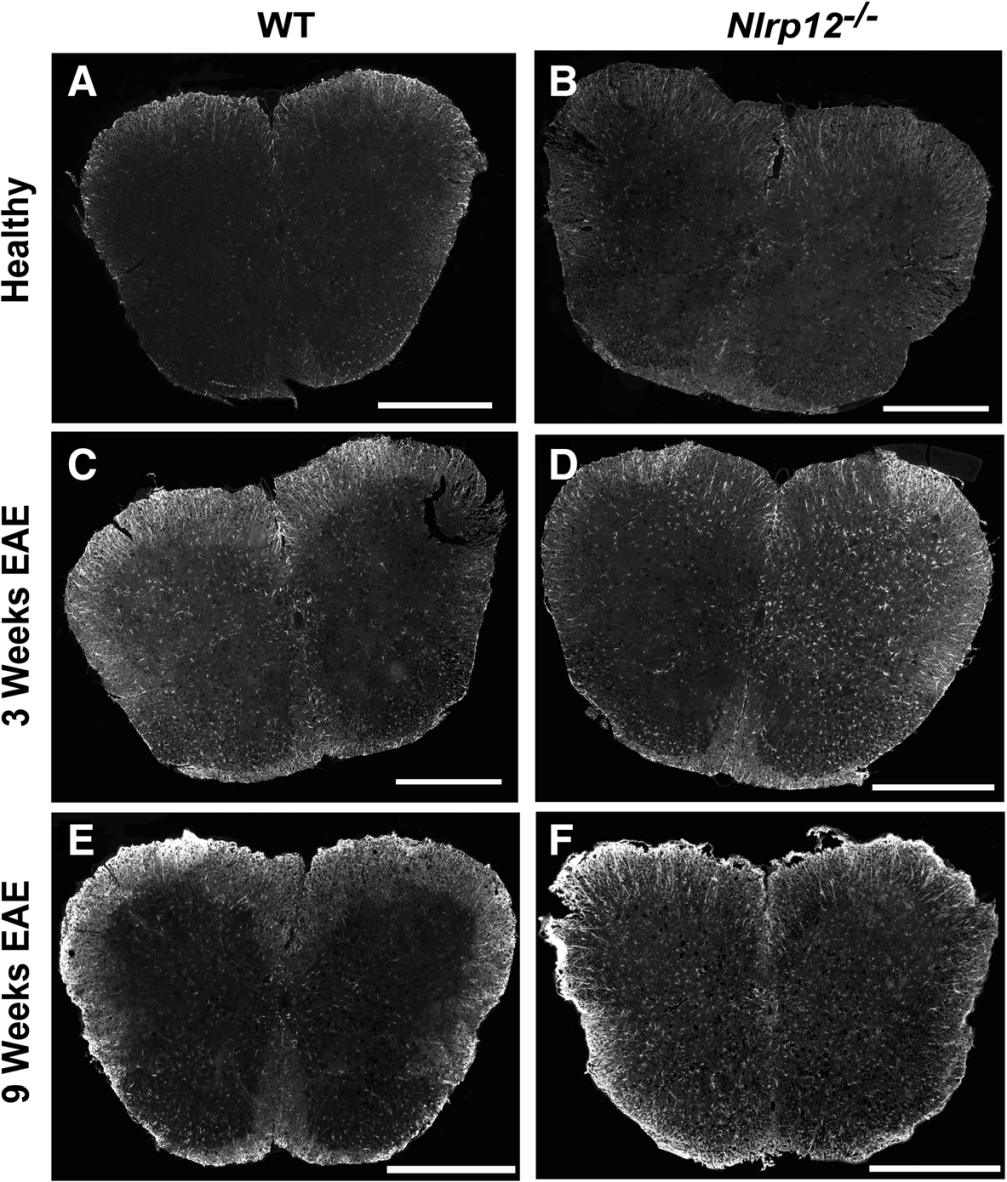
Photomicrograph pictures of the spinal cords stained with GFAP. GFAP staining of the spinal cord, evaluating astrogliosis percentage following EAE induction. **a** WT mice, healthy. **b** *Nlrp12* ^*−/−*^ mice, healthy. **c** WT mice, 3 weeks EAE. **d** *Nlrp12* ^*−/−*^ mice, 3 weeks EAE. **e** WT mice, 9 weeks EAE. **f** *Nlrp12* ^*−/−*^ mice, 9 weeks EAE. Scale bar is 500 μm

**Fig. 4:**
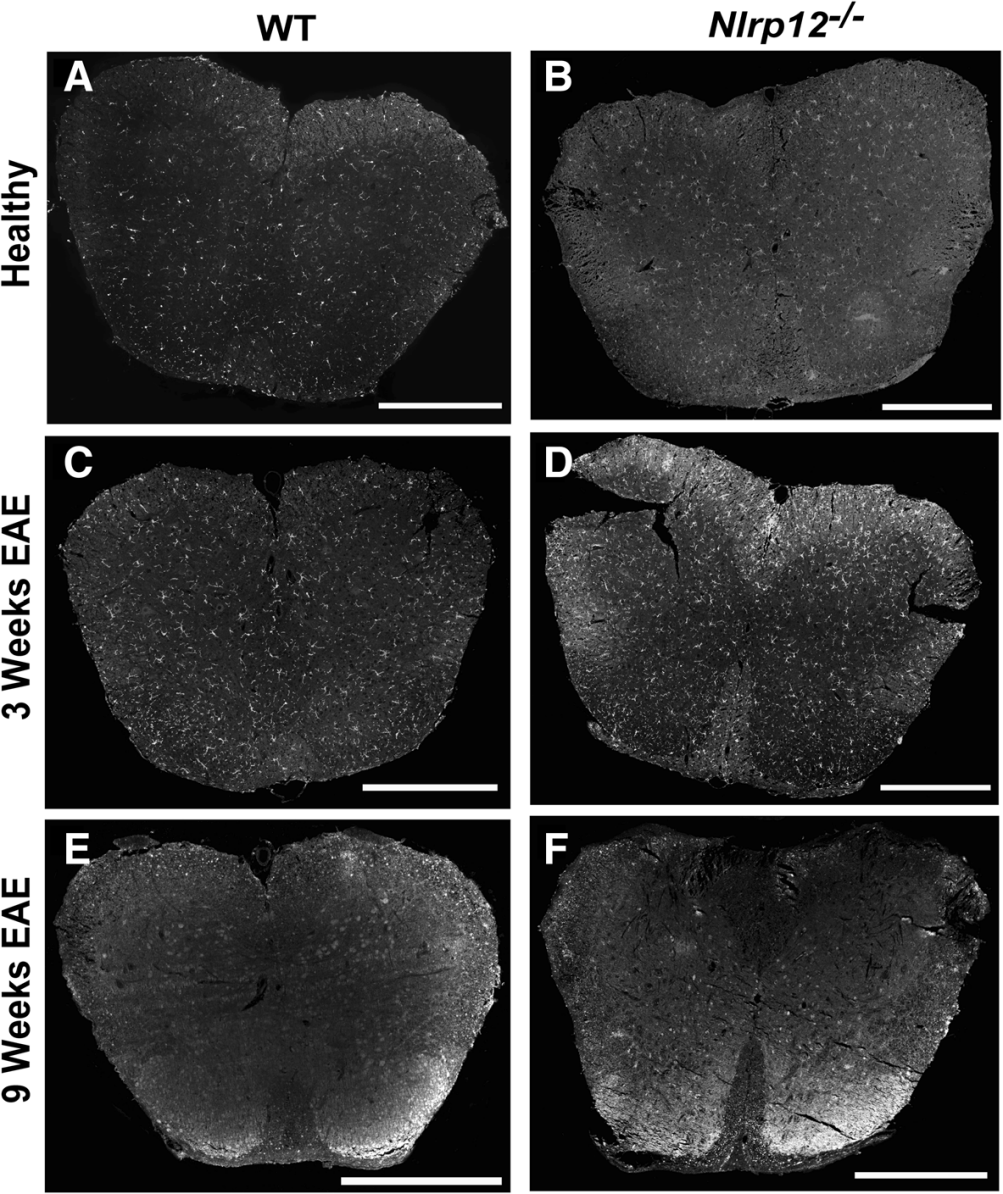
Photomicrograph pictures of the spinal cords stained with Iba1. Iba1 staining of the spinal cord, evaluating microgliosis percentage following EAE induction. **a** WT mice, healthy. **b** *Nlrp12* ^*−/−*^ mice, healthy. **c** WT mice, 3 weeks EAE. **d** *Nlrp12* ^*−/−*^ mice, 3 weeks EAE. **e** WT mice, 9 weeks EAE. **f** *Nlrp12* ^*−/−*^ mice, 9 weeks EAE. Scale bar is 500 μm

**Fig. 5:**
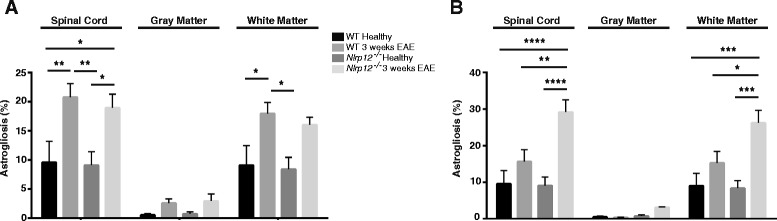
Percent level of astrogliosis following EAE. Percentage of astrogliosis is calculated by the intensity of GFAP staining on total area of the spinal cord. **a** After 3 weeks EAE. **b** After 9 weeks EAE. Results are expressed as mean ± SEM. Statistical significance was accepted at *P < 0.05. Statistical analysis was done by two-way ANOVA followed by Tukey’s multiple comparison test. Each spinal cord was quantified in duplicates and/or triplicates. *n* = 3–4

**Fig. 6:**
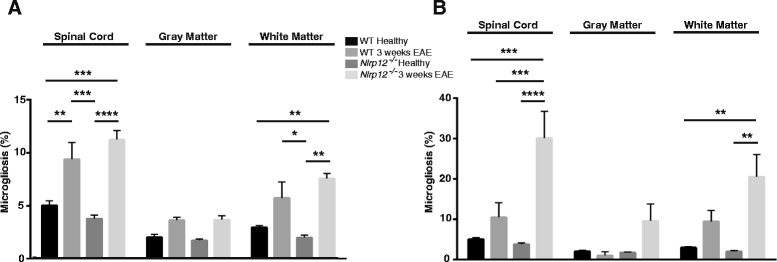
Percent level of microgliosis following EAE. Percentage of microgliosis is calculated by the intensity of Iba1 staining on the total area of the spinal cord. **a** After 3 weeks EAE. **b** After 9 weeks EAE. Results are expressed as mean ± SEM. Statistical significance was accepted at *P < 0.05. Statistical analysis was done by two-way ANOVA followed by Tukey’s multiple comparison test. Each spinal cord was quantified in duplicates and/or triplicates. *n* = 3–5

In response to injury, glial cells become reactive, producing multiple pro-inflammatory proteins, as well as increasing in numbers. GFAP is an intermediate protein expressed and upregulated by astrocytes in response to CNS insults [15]. Moreover, in addition to the secretion of multiple pro-inflammatory proteins, the reactive microglial response can be measured by the extent of upregulation of Iba1 [5]. The spinal cords of the healthy and immunized mice were extracted and stained for GFAP (Fig. 3) and Iba1 (Fig. 4). We observed no significant difference in the percent level of astrogliosis and microgliosis between the healthy WT and healthy *Nlrp12*^*−/−*^ mice. Additionally, we observed no differences in the percentage of neither microgliosis nor astrogliosis between the WT and *Nlrp12*^*−/−*^ mice after 3 weeks of EAE (Figs. 5a, 6a). However, after 9 weeks of disease, the *Nlrp12*^*−/−*^ mice demonstrated a significant increase in the level of astrogliosis (30 % compared to 15 % in WTs) in the white matter (WM) and an observable increase within the gray matter (GM) area of the spinal cord compared to the WT mice (Fig. 5b). The 10 % difference between the *Nlrp12*^*−/−*^ mice and the WT mice occurred within the WM. Similar results were obtained for the level of microgliosis, where the *Nlrp12*^*−/−*^ mice demonstrated increased percentage of Iba1 compared to the WT mice (Fig. 6b). Indeed, the difference of 20 % increase in microgliosis within the spinal cord of the *Nlrp12*^*−/−*^ mice compared to the WT mice was primarily within the WM.

### *Nlrp12* negatively regulates T cell proliferation

**Fig. 7:**
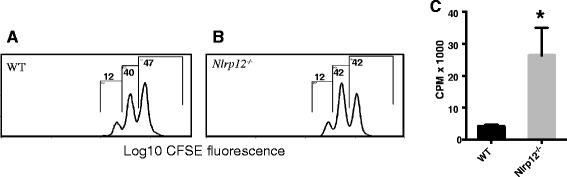
The proliferation of activated T cells from the WT and *Nlrp12* ^*−/−*^ mice in vitro. **a**, **b** CD4^+^ T cells were stained with CFSE and activated with plate-bound anti-CD3/CD28 antibodies stimulation for 3 days. The intensity of CFSE dye in the cells was analyzed by flow cytometry. No significant difference was observed. **c** Purified CD4^+^ T cells from the WT and *Nlrp12* ^*−/−*^ mice were stimulated with anti-CD3/CD28 for 72 h, and the incorporation of 3H-thymidine was measured during the final 18 h of cell culture. Statistical significance was accepted at *P < 0.05. Statistical analysis was done by Mann-Whitney *U* test, *n* = 3–6 per group

We observed higher proliferation in responses to purified CD4^+^ T cells from the *Nlrp12*^*−/−*^ compared to the WT mice (Fig. 7a–c). Interestingly, while pure activation by anti-CD3/CD28 antibodies resulted in the significantly higher proliferative responses in T cells (Fig. 7c) from the *Nlrp12*^−/−^ compared to the WT mice, more physiological activation by splenocytes, although, tended to be higher in T cells from the *Nlrp12*^*−/−*^ mice, did not result in a statistically different proliferation compared to the WT mice (Fig. 7a, b).

### *Nlrp12* deficiency did not affect IL-4 production by activated T cells

**Fig. 8:**
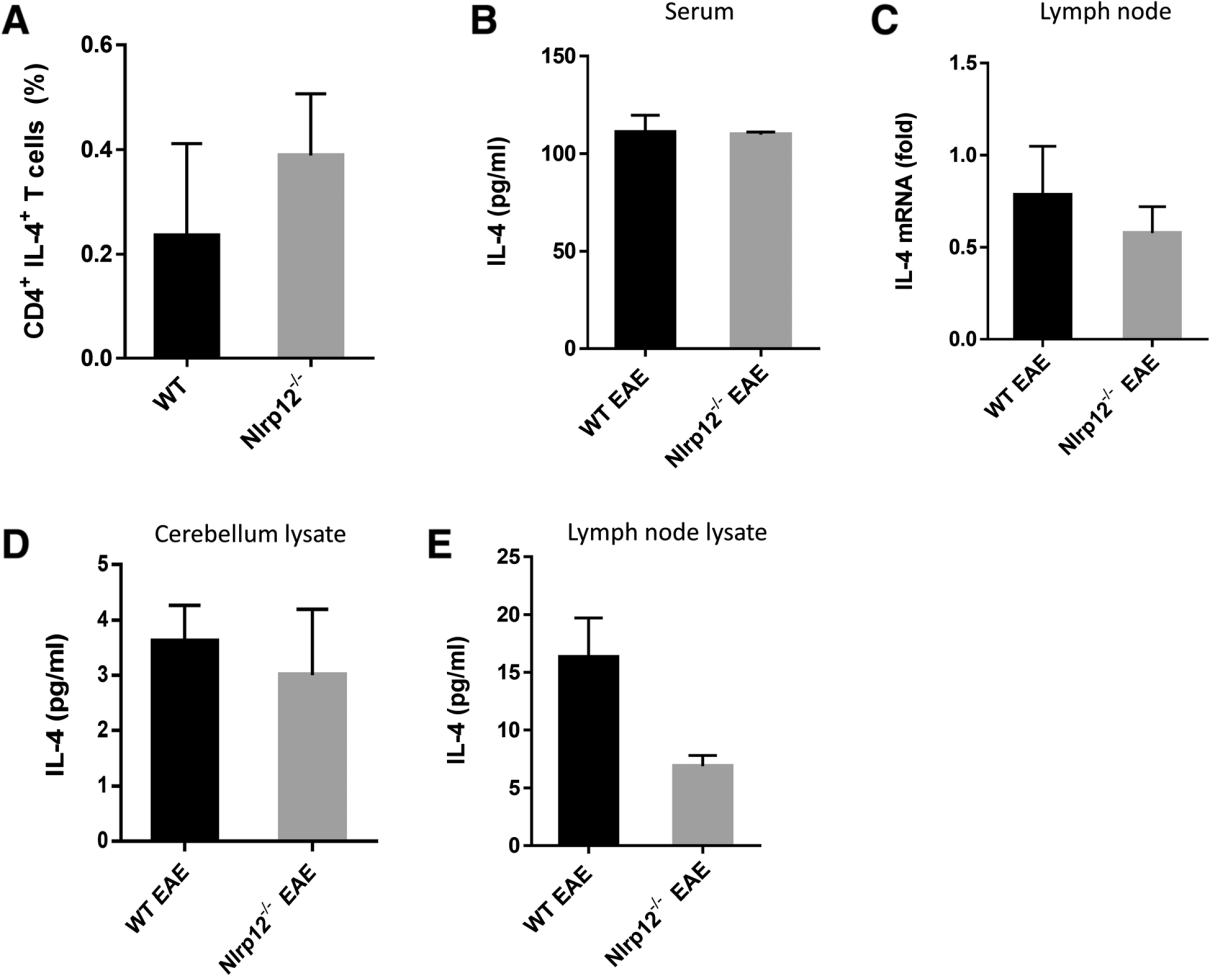
IL-4 production by activated T cells from the WT or *Nlrp12* ^*−/−*^ mice *in vitro* and *in vivo*. **a** CD4^+^ T cells were purified from the lymph nodes and spleens and stimulated with anti-CD3/CD28 antibodies. Intracellular production of IL-4 by activated CD4^+^ T cells was determined using flow cytometry. **b** The levels of IL-4 in serum samples from WT and *Nlrp12* ^*−/−*^ EAE mice, measured by ELISA. **c** The level of IL-4 mRNA in lymph nodes from WT and *Nlrp12* ^*−/−*^ EAE mice, quantified by real-time PCR. **d**, **e** The levels of IL-4 in tissue samples from the WT and *Nlrp12* ^*−/−*^ EAE mice. Cerebellum and lymph node tissues were collected from the mice after 3 weeks of immunization with MOG:CFA. The tissues were homogenized in lysis buffer, and IL-4 levels were measured in tissue lysate by ELISA. Statistical analysis was done by Mann-Whitney *U* test, *n* = 3–6 per group. No significant difference was observed

Differences in T cells proliferation prompted us to verify the levels of IL-4 in the* Nlrp12*^−/−^ mice after EAE induction. We chose to look at IL-4 in light of the recent publication by Lukens et al. that observed that Nlrp12 inhibited Th2 responses. We investigated whether *Nlrp12* deficiency might affect IL-4 production by T cells in EAE mice. As shown in Fig. 8a, no significant difference was detected between the *Nlrp12*^*−/−*^ and WT mice in the percentage of CD4^+^ IL-4^+^ T cells after 3 days of activation with anti-CD3/CD28 antibodies *in vitro*. Consistent with this finding, we did not observe any significant differences in the levels IL-4 in lysates from the lymph nodes of the *Nlrp12*^*−/−*^ or WT EAE mice neither by RT-PCR (Fig. 8c) nor by ELISA (Fig. 8e). Similar observation demonstrated that there was no statistical difference between IL-4 levels in serum (Fig. 8b) and cerebellum (Fig. 8d) from the *Nlrp12*^*−/−*^ EAE mice compared to the WT EAE mice.

### *Nlrp12* deficiency augments expression of pro-inflammatory molecules in the CNS after EAE

**Fig. 9:**
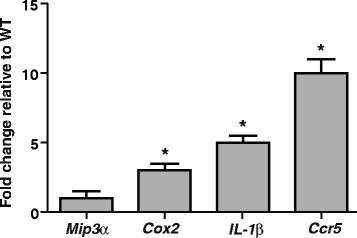
*Nlrp12* deficiency augments expression of pro-inflammatory molecules in the CNS after EAE. Results indicate fold change in mRNA expression of pro-inflammatory proteins in the spinal cords of the *Nlrp12* ^*−/−*^ mice relative to the WT mice. A significant increase in the expression of *Cox2*, *IL-1β*, and *Ccr5* mRNAs and no in the expression of *Mip3α* were observed. Results are expressed as mean ± SD. Statistical significance was accepted at *P < 0.05. Statistical analysis was done using one-way ANOVA followed by Dunet comparison test relative to control. *n* = 5

Looking into the mechanisms of increased inflammation in the *Nlrp12*^*−/−*^mice, we analyzed mRNA expression of pro-inflammatory proteins in the spinal cords of the mice 3 weeks post immunization (Fig. 9). Compared to the WT mice, the *Nlrp12*^*−/−*^ mice demonstrated significantly higher levels of *Cox2* (threefold increase), *IL-1β* (fourfold increase), and *Ccr5* (tenfold increase) mRNA expressions. Although a relative increase in the mRNA expression of *Mip3α* was observed, that difference was not significant. Thus, these results demonstrate that in the absence of *Nlrp12*, the inflammatory response is much more significant.

### *Nlrp12*^*−/−*^ primary microglia express increased levels of reactive species and pro-inflammatory cytokines

**Fig. 10:**
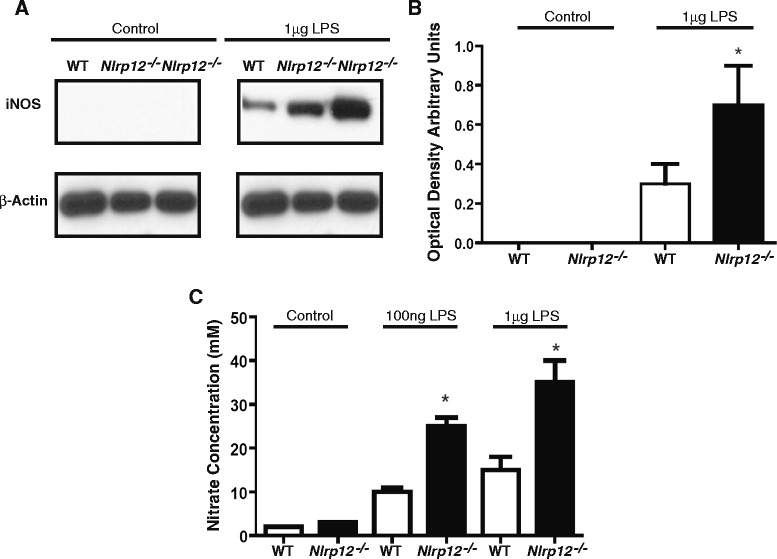
Expression of iNOS in primary microglia cells. Microglia cells (1 × 10^5^) from the *Nlrp12* ^*−/−*^ mice and WT mice were stimulated with 1 ug/mL LPS for 12 h. **a** Western blot analysis. **b** Densitometric analysis of iNOS. **c** Concentration of nitrates using Griess reagent assay. Results are expressed as mean ± SD. Statistical significance was accepted at *P < 0.05. Statistical analysis was done using one-way ANOVA followed by Tukey-Kramer multiple comparison test. *n* = 5

The inflammatory response is an important feature of the innate immunity in the regulation of homeostasis. Inflammation is an innate response that occurs following the encounter of harmful bodies; however, a shift towards anti-inflammatory environment occurs in order to re-establish the balance. Microglia cells play a critical role in this process. Cortices from 1-day-old murine pups were removed, and after 3 weeks in culture, primary microglia cells were separated from astrocytes. Stimulation with bacterial endotoxin lipopolysaccharide (LPS) revealed a significant increase (twofold increase) in the expression of inducible nitric oxide synthase (iNOS), the enzyme responsible for the production of nitric oxide (NO), in *Nlrp12*^*−/−*^ microglia compared to WT microglia (Fig. 10a, b). The supernatants, following LPS stimulation, were further analyzed by Griess reagent assay, and we observed significantly more (2.5-fold increase) nitrates secreted in the media from the microglia of the *Nlrp12*^*−/−*^ mice compared to the WT mice. We additionally observed a dose-response effect (Fig. 10c).

**Fig. 11:**
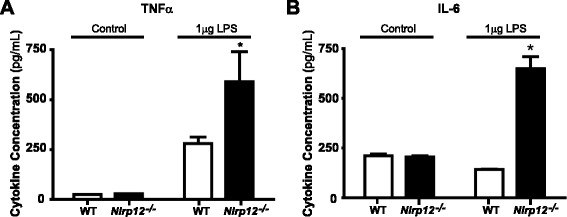
TNF-α and IL-6 concentrations following treatment with LPS in primary microglia cells. Microglia cells (1 × 10^5^) from the *Nlrp12* ^*−/−*^ and WT mice were stimulated with 500 ng/mL LPS for 12 h. **a** ELISA for TNF-α concentration. **b** ELISA for IL-6 concentration. Results are expressed as mean ± SD. Statistical significance was accepted at *P < 0.05. Statistical analysis was done using one-way ANOVA followed by Tukey-Kramer multiple comparison test. *n* = 5

To further characterize microglial response, purified microglia from both genotypes were incubated with 500 ng/mL LPS for 12 h and supernatants were analyzed for the presence of pro-inflammatory cytokines TNFα and IL-6. At basal level, we observed no differences between the WT and *Nlrp12*^*−/−*^ microglia. However, after treatment with LPS, the microglia from the *Nlrp12*^*−/−*^ mice secreted more than twofold increase in TNFα (Fig. 11a) and IL-6 (Fig. 11b) concentrations compared to the WT microglia. Once again demonstrating that in the absence of Nlrp12, the cellular environment is more inflammatory.

## Discussion

The process of inflammation is a fundamental response aimed at protecting the body from foreign and detrimental causes. Neuroinflammation can become harmful if it is unregulated and prolonged. A continuous and persistent response will eventually lead to a chronic state of inflammation, a prominent feature of many neurodegenerative diseases, including MS. NLRP12 is of interest to the study of MS notably due to its restricted expression in cells derived from hematopoietic origins such as monocytes, dendritic cells, and granulocytic cells, and most recently, T cells [16] and its role in attenuating the inflammatory response by interfering in both branches of the NF-κB pathway [9, 17].

To investigate the implication of Nlrp12 in MS, EAE was induced in the WT and in *Nlrp12*^*−/−*^ mice. Our results demonstrated that in mice lacking the *Nlrp12* gene, EAE developed earlier compared to the WT mice, and the *Nlrp12*^*−/−*^ mice showed increased severity throughout the course of the disease. Interestingly, after EAE induction, *Nlrp12* mRNA expression was significantly increased in the WT mice compared to the healthy WTs. These results suggest that Nlrp12 plays an important role in maintaining the level of inflammation and ensuring that a hyper-inflammatory state does not occur. In fact, the expression profile of *Nlrp12* over the course of the disease is suggestive of this regulatory role. Indeed, previous studies have shown that in response to live bacteria such as *M. tuberculosis*, TNFα, and IFNγ, a reduction in *Nlrp12*’s expression is in accordance with an increase in the inflammatory response [18, 19]. Moreover, *Nlrp12*’s over-expression has been previously shown to attenuate the inflammatory response by negatively regulating the NF-κB pathways [17]. A study conducted by Shami et al. demonstrated that the expression of *Nlrp12* is increased in response to nitric oxide [20]. NO is a reactive molecule that is produced in iNOS at sites of inflammation in MS, and it is involved in lesion development [21]. As T cell responses play a crucial role in the development of EAE [12], we evaluated purified CD4 T cell proliferative response after CD3/CD28 activation and we saw significantly elevated proliferation of T cells from the *Nlrp12*^−/−^ mice. We then evaluated T cells proliferation using recall response 10 days after EAE induction by stimulating purified CD4 T cells with MOG peptide in the presence of splenocytes. We observed the tendency of T cells from the *Nlrp12*^−/−^ mice for a higher proliferation rate; however, these differences never reached a statistical significance. These results are similar to those published by Lukens et al. [16], where authors observed that pure anti-CD3/CD 28 activation resulted in significantly higher proliferation in T cells from *Nlrp12*^*−/−*^ mice, while in the presence of splenocytes, differences between T cell proliferation of different genotypes were greatly reduced. In this work, the authors propose that the cell autonomous effect of *Nlrp12* in T cells shifts T cell differentiations to a Th2-IL-4 producing phenotype [16].

We measured the concentration of IL-4 in the serum, lymph nodes, and brain samples from the WT and from *Nlrp12*^*−/−*^ mice at 3 weeks after EAE and did not find any differences in the expression of IL-4. These results are consistent with our observation that there were no differences in percentage of IL-4 producing cells and that results from experiments in complex cellular interaction at the tissue level in vivo can be different from results of clean anti-CD3/CD28 activation in vitro. Till today, no exact mechanism has been described that explains *Nlrp12* activity in different cell types. *Nlrp12* has been shown to inhibit classical and alternative pathways of NF-κB in different cell types and different stimulations; for extensive review, please read Tuncer et al. [9]. In light of these controversies, the different KO strategies to remove *Nlrp12* may have produced an uncontrolled variable that resulted in different phenotypes [22, 23]. Future studies should address these differences.

In our studies we observed that *Nlrp12*^*−/−*^ mice demonstrated more severe course of EAE according to classical evaluation of clinical scores, while in the work by Lukens and co-workers, the authors noted appearances of the atypical EAE. These results are intriguing, as overall effect of *Nlrp12* on the EAE pathology was similar to our observations. Furthermore, EAE is a well-characterized and the most widely used mouse model to study MS [13]. It exhibits the main features of MS pathology such as inflammation, destruction of myelin, and reactive gliosis. Moreover, many of the current therapies for MS, such as Tysabri were developed following EAE studies [24]. However, it is important to note that the evaluations of clinical scores are subjective. In our studies, we did not measure the degree of atypical EAE as there is no quantifiable scale to evaluate this pathology. Observing video clips published by Lukens et al. (supplemental materials), we can tell that *Nlrp12* mouse was severely compromised and had impaired righting reflex, which suggests severe weakness/paralysis of the hind limbs as well as paralysis of the trunk muscles.

To further elucidate how *Nlrp12* is playing a protective role in the disease, the spinal cords of both the WT and *Nlrp12*^*−/−*^ mice were analyzed for the expression of genes implicated in EAE as well as in MS. Our results demonstrated a significant increase in the mRNA expression of *Cox-2*, *IL-1β*, and *Ccr5* genes in the *Nlrp12*^*−/−*^ mice compared to the WT mice, suggesting a protective role played by *Nlrp12* in EAE at the level of pro-inflammatory gene expression. The increase in expression of pro-inflammatory molecules in *Nlrp12*-deficient phenotype has been demonstrated by multiple studies [22, 25].

Next, we demonstrated that *Nlrp12* inhibits inflammation during EAE at the level of microglia. We showed that *Nlrp12* deficiency augments pro-inflammatory microglial phenotypes by using purified primary microglia cells from the WT and *Nlrp12*^*−/−*^ mice. Consistent with our in vivo observation, stimulation of microglia with LPS resulted in a significant increase of iNOS expression, NO, TNFα, and IL-6 secretion from the *Nlrp12*^*−/−*^ microglia cells compared to the WT microglia. These results are consistent with the suppressive role of *Nlrp12* in cells of myeloid origin [26]. A report by Lukens et al. also found increased inflammatory response in the CNS tissue of *Nlrp12*^*−/−*^ mice compared to WT controls, although, microglia responses per se were not verified. Furthermore, the notion of inhibitory NLRs is not new. Similar to our results, stimulation of primary *Nlrx1*^*−/−*^ microglia cells revealed a significant increase in the pro-inflammatory response, thus, showing a suppressive role for Nlrx1 in microglial activation [27].

The roles of microglia and astrocytes are well defined in the pathology of MS. Previous studies on Nlrp3 have demonstrated that the absence of this receptor results in better disease outcome and reduced gliosis following EAE [28]. The spinal cords of the *Nlrp12*^*−/−*^ mice and WT mice were stained with Iba1 and GFAP in order to assess the extent of microgliosis and astrogliosis, respectively. Surprisingly, no differences in the percentage of gliosis were observed between the two genotypes at the third week; however, after 9 weeks, the *Nlrp12*^*−/−*^ mice demonstrated significantly increased gliosis compared to the WT mice. Additionally, in both genotypes, the majority of gliosis occurs within the white matter area of the spinal cord. Although a quantitative difference was not observed at the third week, in vitro study suggests qualitative changes in microglia activation. Indeed upon LPS stimulation, microglia from the *Nlrp12*^*−/−*^ mice released significantly more pro-inflammatory mediators. Furthermore, the remarkable increase in *Ccr5* mRNA expression observed in the *Nlrp12*^*−/−*^ mice suggests that Nlrp12 may be playing a crucial role in the influx of inflammatory infiltrates. CCR5 is a chemokine receptor that is expressed primarily by monocytes, macrophages, effector T cells, immature dendritic cells, and NK cells [29]. Moreover, previous studies in both animal and in MS patients have demonstrated the upregulation of CCR5 in inflammatory lesions [30–32]. Also, a chronic over-expression of IL-1β has been shown to result in the disruption of the blood-brain barrier (BBB) and in the infiltration of leukocytes such as macrophages, DCs, and neutrophils [33, 34]. Thus, the increase of *Ccr5* and *IL-1β* mRNA in the spinal cords of the *Nlrp12*^*−/−*^ compared to the WT mice during EAE supports the notion of an increased influx of inflammatory cells in these mice. In fact, the entry of pro-inflammatory leukocytes into the CNS is an early phenomenon capable of initiating events that result in BBB disruption and neuroinflammation [35]. Interestingly, previous studies have demonstrated a reduction in inflammatory infiltrates within the CNS in EAE-induced *Nlrp3*^*−/−*^ mice, where Nlrp3 was shown to play an inflammatory role by inducing immune cell migration whereas, our results suggest that *Nlrp12* plays a protective role by maintaining the level of inflammatory influx [36, 37]. Thus, future studies should focus on evaluating in details the presence of inflammatory infiltrates in order to clarify the driving force responsible for the differences observed between WT and *Nlrp12*^*−/−*^ mice.

## Conclusion

The study of NLRs and their functions has been mainly studied in the context of host and pathogen interactions. Their role in mediating the inflammatory response is well recognized, while their role in other diseases is an emerging field. Recent reports suggest that Nlrs may play a detrimental as well as beneficial role in the progression of EAE. For example, Nod1, Nod2, and Nlrp3 augment inflammation and T cell responses that lead to increased EAE severity. On the other hand, the expression of *Nlrp12* and *Nlrx1* inhibits the expression of pro-inflammatory genes, suppressing inflammation and reducing the severity of EAE [27]. In many neurodegenerative diseases the regulation of neuro-inflammatory responses is a key target for therapeutic interventions. Numerous studies have focused on the role and contribution of T- and B-lymphocytic responses in MS, and much of the pathophysiology of MS has gravitated around the adaptive branch of the immune system. The implication of the adaptive immune response is undeniable in this disorder, given that the primary cause of damages in the nervous system of MS patients is due to CNS inflammation, where CD4^+^ autoreactive T cells primarily react to myelin epitope, enter the CNS, and result in the destruction of myelin [3]. At this stage, we are not excluding the role of Nlrp12 in T cell responses during EAE. However, it is vital to understand the underlying cause of the inflammatory process in MS. Thus, it is important to focus on the innate immune response, since in essence, inflammation is a response of innate immunity [38]. Thus our findings that Nlrp12 plays a role in microglia activation during EAE may help find the mechanism that regulates CNS specific inflammation.

## Abbreviations

APCs: antigen-presenting cells
BBB: blood-brain barrier
CCL22: CC chemokine ligand 22
CCL5: CC chemokine ligand 5
CCR5: CC chemokine receptor 5
CFA: complete Freud’s adjuvant
CFSE: carboxyfluorescein diacetate succinimidyl ester
CNS: central nervous system
COX2: cyclooxygenase 2
DCs: Dendritic cells
EAE: experimental autoimmune encephalomyelitis
GFAP: glial fibrillary acidic protein
GM: gray matter
Iba1: ionized calcium binding adaptor molecule 1
IFNγ: interferon gamma
IL-1β: interleukin-1-beta
IL-4: interleukin-4
IL-6: interleukin-6
iNOS: inducible nitric oxide synthase
LPS: lipopolysaccharide
LRR: leucine-rich repeat
MHC class II: major histocompatibility complex class II
MIP1α: macrophage inhibitory protein 1 alpha
MIP3α: macrophage inhibitory protein 3 alpha
MOG: myelin oligodendrocyte glycoprotein
mRNA: messenger ribonucleic acid
MS: multiple sclerosis
NACHT: NAIP (neuronal apoptosis inhibitor protein), C2TA (MHC class 2 transcription activator), HET-E (incompatibility locus protein from Podospora anserina), and TP1 (telomerase-associated protein)
NF-κB: nuclear factor-kappa B
NK: natural killer
NLR: nod-like receptors
NO: nitric oxide
OVA: ovalbumin
PAMP: pathogen-associated molecular patterns
PRR: pathogen-recognition receptors
Th2: T helper cell 2
TNFα: tumor necrosis factor alpha
WM: white matter
WT: wild type
